# Staying awake – a genetic region that hinders α_2_ adrenergic receptor agonist-induced sleep

**DOI:** 10.1111/ejn.12570

**Published:** 2014-03-27

**Authors:** Cigdem Gelegen, Thomas C Gent, Valentina Ferretti, Zhe Zhang, Raquel Yustos, Fei Lan, Qianzi Yang, Dorothy W U Overington, Alexei L Vyssotski, Hein A van Lith, William Wisden, Nicholas P Franks

**Affiliations:** 1 Department of Life Sciences, Imperial College LondonSouth Kensington, London, SW7 2AZ, UK; 2 Institute of Neuroinformatics, University of Zurich/ETH ZurichZurich, Switzerland; 3 Division of Animal Welfare & Laboratory Animal Science, Department of Animals in Science and Society, Faculty of Veterinary Medicine, Utrecht UniversityUtrecht, The Netherlands; 4 Brain Center Rudolf MagnusUtrecht, The Netherlands

**Keywords:** alpha2a adrenergic receptor, sedation, sleep, wakefulness

## Abstract

How external stimuli prevent the onset of sleep has been little studied. This is usually considered to be a non-specific type of phenomenon. However, the hypnotic drug dexmedetomidine, an agonist at α_2_ adrenergic receptors, has unusual properties that make it useful for investigating this question. Dexmedetomidine is considered to produce an ‘arousable’ sleep-like state, so that patients or animals given dexmedetomidine become alert following modest stimulation. We hypothesized that it might be more difficult to make mice unconscious with dexmedetomidine if there was a sufficient external stimulus. Employing a motorized rotating cylinder, which provided a continuous and controlled arousal stimulus, we quantitatively measured the ability of such a stimulus to prevent dexmedetomidine loss of righting reflex in two inbred strains of mice (C57BL/6 and 129X1). We found that whereas the C57BL/6 strain required a strong stimulus to prevent dexmedetomidine-induced hypnosis, the 129X1 strain stayed awake even with minimal stimuli. Remarkably, this could be calibrated as a simple threshold trait, i.e. a binary ‘yes–no’ response, which after crossing the two mouse strains behaved as a dominant-like trait. We carried out a genome-wide linkage analysis on the F_2_ progeny to determine if the ability of a stimulus to prevent dexmedetomidine hypnosis could be mapped to one or more chromosomal regions. We identified a locus on chromosome 4 with an associated Logarithm of Odds score exceeding the pre-established threshold level. These results show that complex traits, such as the ability of a stimulus to reverse drug-induced hypnosis, may have precise genetic determinants.

## Introduction

How do irritations in the environment, such as noise, or other general disturbances stop us falling asleep? To get at this question mechanistically, we decided to use a drug, the adrenergic agonist dexmedetomidine, which induces a type of hypnosis resembling natural sleep (Kamibayashi & Maze, 2000). We wanted to see if falling asleep after administration of this drug could be antagonized by external stimuli. The adrenergic system in the brain is intimately involved in regulating the sleep–wake cycle (Saper *et al*., 2010; Berridge *et al*., 2012; Schmeichel & Berridge, 2013; Szabadi, 2013). There are three classes of adrenergic receptors expressed in the brain: α_1_, α_2_ and β (Philipp & Hein, 2004; Berridge *et al*., 2012; Szabadi, 2013). Activation of the α_1_ and β receptor classes produces excitation (Schmeichel & Berridge, 2013), whereas activation of inhibitory α_2A_ receptors with agonists such as clonidine, xylazine or dexmedetomidine, induces hypnosis (Drew *et al*., 1979). Of these three drugs, dexmedetomidine is the most selective agonist at α_2_ adrenergic receptors, which are essential for its sedative and hypnotic actions (Maze & Regan, 1991; Lakhlani *et al*., 1997; Kamibayashi & Maze, 2000). Deletion or global knockdown of the gene (*Adra2a*) that codes for the α_2A_ adrenergic receptor in mice abolishes the ability of dexmedetomidine to induce loss of righting reflex (LORR; Lakhlani *et al*., 1997; Tan *et al*., 2002; Gilsbach *et al*., 2009), which is used as the animal surrogate for loss of consciousness (Franks, 2008). What makes dexmedetomidine unusual is that it produces an ‘arousable’ anaesthetic state. That is to say, patients administered dexmedetomidine become alert and can respond to commands following modest stimulation such as shaking (Kamibayashi & Maze, 2000; Venn & Grounds, 2001). Indeed, hypnosis induced with dexmedetomidine appears to be a ‘sleep-like’ state, with the electroencephalogram (EEG) resembling that of natural, non-rapid eye movement (NREM) sleep, rather than conventional anaesthesia (Mason *et al*., 2009).

Because the expression pattern of the *Adra2a* gene in the brain is quite restricted (Nicholas *et al*., 1993; Wang *et al*., 1996), it might be possible to not only determine which downstream neuronal pathways are recruited by dexmedetomidine to induce sleep but also, conversely, which pathways might be involved in preventing this sleep-like state. This in turn might give novel insight into natural sleep circuitry (Franks, 2008). To this end, we devised a novel protocol to measure the ability of a stimulus to hinder sleep in the presence of dexmedetomidine. We found that whereas the C57BL/6 strain resisted dexmedetomidine-induced LORR only when given a sufficiently strong arousal stimulus, another strain, 129X1, could be kept awake with just minimal stimulation. Encouraged by how mouse breeding studies have identified hereditary components of the EEG and other sleep parameters (Franken *et al*., 1998; Tafti *et al*., 2003; Maret *et al*., 2005; Andretic *et al*., 2008; Winrow *et al*., 2009), we decided to map the genetic region underlying this ‘sleep-hindering’ trait by undertaking linkage analysis on the F_2_ progeny. We identified a locus that influences the ability or, conversely, inability, to fall asleep, as mimicked by dexmedetomidine administration, in the face of an intruding external stimulus.

## Materials and methods

### Mice

All experiments were performed in accordance with the UK Home Office Animal Procedures Act (1986), and all procedures were approved by the Imperial College Ethical Review Committee. Inbred mice, C57BL/6 (C57BL/6JOlaHsd) and 129X1 (129X1/SvJ), were purchased from Harlan Laboratories UK (Blackthorn, Bicester, UK) and The Jackson Laboratory (Bar Harbor, ME, USA), respectively. A total of 141 mice were used.

### Arousal assay

Dexmedetomidine (40 μg/mL; Tocris, Bristol, UK) or propofol (10 mg/mL; Fresenius Kabi, Runcorn, UK) was delivered intraperitoneally (i.p.), and animals were placed in a custom-built apparatus consisting of a continuously rotating transparent cylinder driven by a variable-speed motor. During 15 min, animals were scored as positive for LORR if they had rolled onto their backs in the rotating cylinder and made no obvious attempt to right themselves. The assay was repeated at different rotation speeds. Although LORR is a crude surrogate for loss of consciousness, it is reproducible, precise and widely used, and anaesthetic concentrations for LORR in rodents correlate well with concentrations that induce loss of consciousness in humans (Franks, 2008). ED_50_ values and their associated errors were calculated using the method of Waud (1972) for the analysis of quantal dose–response data. The arousal assay determines the amount of stimulus (rotation speed) required to reverse LORR.

### Rotarod assay

Mice were trained on a rotarod (Med Associates, St Albans, VT, USA) essentially as described previously (Korpi *et al*., 1999). For the pre-training, animals were placed on the rotarod at rest and the rod was accelerated at 0.5 rpm. Initially, C57BL/6 mice fell off at 25.1 ± 2.3 rpm (mean ± SEM), whereas 129X1 mice fell off at 13.7 ± 4.2 rpm (*P *< 0.01, unpaired Student's *t*-test). The rotarod training schedule consisted of daily 10-min periods of constant speed. For the post-training performance on the rotarod, the two strains of mice achieved equal performance – C57BL/6 mice fell off at 25.2 ± 3.8 rpm and 129X1 mice at 24.4 ± 3.1 rpm (*P *> 0.1). Trained mice were then given dexmedetomidine i.p. as above.

### EEG recording

Mice were chronically implanted with skull screw electrodes (−1.5 mm Bregma, + 1.5 mm midline – first recording electrode; + 1.5 mm Bregma, −1.5 mm midline – second recording electrode; −1 mm Lambda, 0 mm midline – reference electrode) to measure cortical EEG, and the electrical signals were recorded on a wireless electronic recording device (Neurologger 2, constructed and supplied by A.L.V.) as described previously (Pang *et al*., 2009; Vyssotski *et al*., 2009; Zecharia *et al*., 2012). Male mice were instrumented with the EEG electrodes at the age of 10 ± 2 weeks. The animals were allowed 2 weeks to recover from surgery before the experiments were performed. Four data channels could be recorded at a sampling rate of 200 Hz and were low-pass filtered with a cut-off at 1 Hz (−3 db). The EEG data recorded by the Neurologger were downloaded to a PC, and waveforms were visualized using Spike2 software (Cambridge Electronic Design, Cambridge, UK) or MATLAB (MathWorks, Cambridge, UK). EEG data were analysed using either Morlet wavelets to follow changes in time as previously described (Pang *et al*., 2009), or using Fourier transforms to average power spectra over time. Fourier transforms were averaged over 60 s for both the control EEG and EEG in the presence of dexmedetomidine. The data for the controls were taken 5 min before drug administration, and the data with dexmedetomidine were taken 10 min after LORR.

### Comparing *Adra2a* gene sequences between mouse strains

The *Adra2a* coding sequence was amplified from genomic DNA extracted from ear punches (*n *= 3 for each strain). The primers were sense, 130 bp upstream of the ATG codon: 5′-GCTCCCTGCGGCCCTCTTC-3′; and antisense, 44 bp downstream of the stop codon: 5′-CCTGCGTCTGACCATTGTCTG-3′. The 1571-bp products were sent for sequencing at Eurofins MWG Operon (Ebersberg, Germany).

### Measuring *Adra2a* mRNA levels

The *Adra2a* mRNA levels were determined in the septum, neocortex and locus coeruleus in male mice by reverse transcriptase-polymerase chain reaction (PCR; *n *= 7 for each strain; Taqman® RNA-to-CT™1-Step kit; Applied Biosystems, Foster City, USA). Brains were removed and 1-mm tissue punches were prepared using 1-mm interval mouse brain matrix (Zivic Instruments, Pittsburgh, PA, USA) and 1-mm core diameter hollow needles. Total RNA was extracted from frozen tissues using TRIzol. Real-time quantitative PCR was performed on an ABI StepOne Plus Real Time PCR system (Applied Biosystems, Foster City, USA). Data were evaluated with SDS 2.1 software, using the Comparative *C*_t_ method (ΔΔ*C*_t_) to measure gene expression. Relative expression of the *Adra2a* gene was determined by comparing its expression with that of hypoxanthine phosphoribosyl-transferase (*Hprt*).

### *In situ* hybridization

We used ^33^P-labelled oligodeoxyribonucleotides as described previously (Wisden & Morris, 1994). The probe sequence used was: 5′-CTCACACGATGCGTTTTCTGTCCCCACGGCAGAGGATCTTCTTGAAGG-3′.

### Genotyping the F_2_ population

To determine if our observed phenotype could be mapped to a particular locus on the genome, we first identified a number of microsatellite markers, spaced across the genome, so that we could use Quantitative Trait Locus (QTL) analysis (see below) to see which markers segregated with the phenotype. The microsatellite markers that differed (i.e. were polymorphic) between the two parental inbred strains were obtained from the Mouse Microsatellite Database of Japan (http://www.shigen.nig.ac.jp) and from Prows *et al*. (2007). Our studies on adult mice from the F_1_ generation derived from reciprocal mating of C57BL/6 females to 129X1 males and of 129X1 females to C57BL/6 males indicated that the ability to reverse dexmedetomidine LORR by a stimulus was not sex-linked. Therefore, we did not include sex chromosome markers. The average distance between consecutive markers was ∼ 11 cM (see Figure S1 in Supporting Information for the genetic map of the markers). Sequences of primers for the amplification of the microsatellite markers were obtained from the Mouse Genome Informatics database (http://www.informatics.jax.org). PCR conditions were optimized for each primer using genomic DNAs from the two grandparent inbred strains.

### QTL analyses and other statistical methods

A QTL is the most likely position (= locus) on the genome that is associated with genetic differences for a (complex) phenotypic trait (Kas *et al*., 2009; Hart & Ruvolo, 2012; Hessel *et al*., 2012). QTL analysis is a statistical method that correlates trait variation with DNA variation in experimental (or natural) populations. The correlation between the trait and the DNA variation is estimated and plotted along the genome to produce a QTL profile. The approach can be applied to both continuous and binary phenotypes, with the latter sometimes being referred to as Binary Trait Analysis (Hart & Ruvolo, 2012). Our phenotype, the ability of a stimulus to prevent dexmedetomidine-induced LORR, was scored as a ‘yes–no’ binary trait (at the 3.5 rpm rotation speed of the cylinder, the mouse either lost its righting reflex following dexmedetomidine injection or it did not). QTL mapping was performed on 55 male F_2_ progeny using R/qtl software (Broman & Sen, 2009), and Logarithm of Odds (LOD; a statistical measure of the likelihood of linkage) scores were calculated across the genome using a ‘binary’ model (Broman & Sen, 2009). LOD scores were determined by interval mapping, which calculates the probability of linkage at each position in the genome by making use of the two markers, which flank each position. Linkage is considered significant if the LOD score exceeds a pre-established statistical threshold. The genome-wide LOD score threshold level (at a confidence level of 0.05) was determined through permutation tests (random shuffling of genotypes with phenotypes; 10 000 permutations). In separate calculations, QTL analysis was also performed by Multiple-QTL-Mapping (MQM), which is more powerful than traditional parametric interval mapping (Arends *et al*., 2010). Through this method the search for a single QTL is enhanced by taking into account in the statistical model QTLs elsewhere in the genome, which is done by using as cofactors DNA markers closely linked to these QTLs. This way, the markers used as cofactors are expected to eliminate the major part of the variation induced by nearby QTLs and as a result the power of the QTL analysis is enhanced. Two markers (one on chromosome 9 and one on chromosome 13) were used as cofactors in the MQM module. The confidence interval (in bp) for the region on chromosome 4 linked to the arousability was determined using the ‘LOD drop-off’ method where minus 1 LOD support interval is constructed by taking the two positions left and right of the maximum value in the LOD profile that have a LOD value of 1 less than this maximum; the two positions are estimated using linear interpolation calculations.

Student's *t*-test was used to test for significant differences in rotarod performance between the two mouse strains and in expression levels of *Adra2a* mRNA in different brain regions assessed by real-time PCR.

## Results

### Dexmedetomidine induces sedation with equal potency in 129X1 and C57BL/6 mice strains

**Figure 1:**
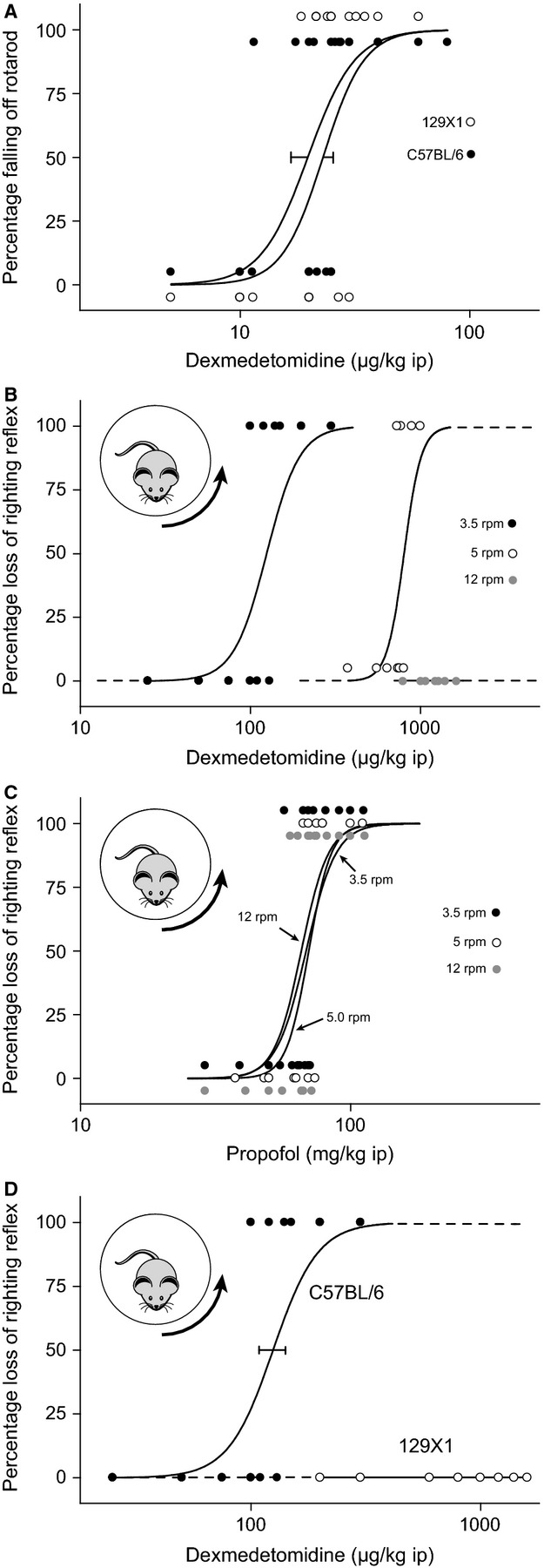
The ability of a stimulus to prevent dexmedetomidine-induced loss of righting reflex (LORR) differs with mouse strain. (A) At low concentrations, dexmedetomidine is equally potent in causing sedation/ataxia in both C57BL/6 (closed circle) and 129X1 (open circle) mice in a Rotarod assay (Wulff *et al*., 2007). (B) The dose of dexmedetomidine required to cause LORR in C57BL/6 mice increases with increasing arousal stimulus (closed circle: 3.5 rpm, *n* = 7; open circle: 5 rpm, *n* = 10; grey circle: 12 rpm, *n *= 7). (C) The dose of propofol required to cause LORR in C57BL/6 mice does not change with increasing arousal stimulus (closed circle: 3.5 rpm, *n* = 18; open circle: 5 rpm, *n* = 14; grey circle: 12 rpm, *n *= 17). (D) With modest stimulation (3.5 rpm), dexmedetomidine causes LORR in C57BL/6 (closed circle: 3.5 rpm, *n* = 7) mice (data from B replotted for comparison) but not in 129X1 (open circle: *n *= 11) mice. Each data point represents a mouse at a given dose, and the dose–response curves were calculated from these binary data using the method of Waud (1972).

It is well established that with increasing dose the α_2_ adrenergic agonist dexmedetomidine induces first sedation and then loss of consciousness in animals and humans (Kamibayashi & Maze, 2000). As expected, we found that mice from both the 129X1 and C57BL/6 strains appeared heavily sedated at low doses of dexmedetomidine (50 μg/kg) but had not lost their righting reflex. They had generally reduced movements, and a low body and head posture. We quantified any differences in sedation at low concentrations of dexmedetomidine using the Rotarod assay. Animals were trained daily and both strains achieved the same level of competence on the Rotarod after several days training. Dexmedetomidine (5–80 μg/kg) had a similar ability to induce sedation/ataxia in both trained 129X1 and C57BL/6 mice, as assessed with the Rotarod assay (Fig.1A), suggesting that α_2A_ adrenergic receptors and their associated signalling mechanisms are working similarly in both strains.

### Dexmedetomidine-induced LORR can be prevented with a sufficient stimulus

As reported previously by others (Lakhlani *et al*., 1997; Gilsbach *et al*., 2009), we also found that at higher than sedative concentrations (400 μg/kg) dexmedetomidine was effective at causing unstimulated LORR in both C57BL/6 and 129X1 mice (data not shown). We next measured how well the mice could, after being administered dexmedetomidine, maintain wakefulness in the presence of a stimulus. Mice were injected with variable concentrations (25–2000 μg/kg) of dexmedetomidine (i.p.) and then placed in a slowly rotating Plexiglas cylinder and the EEG simultaneously recorded using a non-tethered recording system (Neurologger 2). In this assay, mice are forced to continuously adjust their posture (Fig.1B); LORR is taken to be the point where the mouse passively rolls on to the side of the cylinder and can no longer adjust its gait to the moving surface of the cylinder. During 15 min the animals were scored for LORR.

We found that at a relatively low rotation speed of 3.5 rpm dexmedetomidine induced LORR in C57BL/6 mice with an ED_50_ of 125 ± 16 μg/kg (Fig.1B; see Data S1, videos S1 and S2). However, if the arousal stimulus to the mouse was increased by increasing the rotation speed of the cylinder, a higher dose of dexmedetomidine was required to induce LORR. At 5 rpm, the ED_50_ was 801 ± 114 μg/kg. At the highest rotation speed (12 rpm), doses up to 2000 μg/kg failed to induce LORR. Therefore, this assay is also a quantitative model for arousability, analogous to how patients who have been given dexmedetomidine can be woken up.

In contrast, and as expected, the arousal intensity had no effect on the ability of the general anaesthetic propofol to induce LORR – propofol (30–140 mg/kg) induced a dose-dependent LORR that did not change significantly with the rotation speed of the cylinder (Fig.1C).

### The ability of a stimulus to counteract dexmedetomidine-induced LORR depends on mouse strain

At a cylinder rotation speed of 3.5 rpm we found that dexmedetomidine, even at doses up to 2 mg/kg, could not induce LORR in 129X1 mice (see Data S1, videos S3 and S4), but C57BL/6 mice were sensitive (Fig.1D; see Data S1, videos S1 and S2). At this speed the assay showed a binary ‘yes–no’ sensitivity between the two strains. The effect was independent of sex of the mice.

### Differences between the mouse strains in the ability of a stimulus to prevent dexmedetomidine-induced LORR is reflected in their EEGs

**Figure 2:**
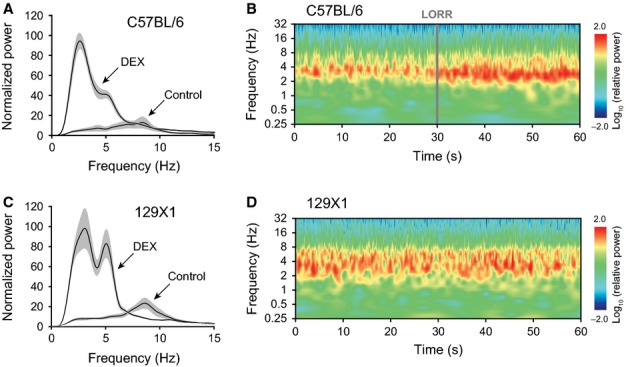
The difference in dexmedetomidine sensitivity between mouse strains is reflected in the EEG. (A) Fourier transform power spectra for controls, and 10 min following dexmedetomidine administration in C57BL/6 mice (*n *= 6). Dexmedetomidine induces a dominant band at delta frequencies and a shoulder at theta frequencies. The grey areas show the SEMs. (B) The averaged (*n *= 6) Morlet Wavelet power spectrum shows an abrupt change in power and frequency of delta oscillations when 400 μg/kg dexmedetomidine causes a loss of righting reflex (LORR) in C57BL/6 mice in the rotating cylinder. The spectra have been aligned at LORR. (C) Fourier transform power spectra for controls, and 10 min following dexmedetomidine administration in 129X1 mice (*n *= 4). Dexmedetomidine induces bands of both delta and theta frequencies. (D) The averaged (*n *= 4) Morlet Wavelet power spectrum shows unchanged delta/theta oscillations in 129X1 mice during the same time period following injection of dexmedetomidine but no LORR in the rotating cylinder.

Animals were chronically implanted with EEG electrodes and given 400 μg/kg dexmedetomidine (i.p.). The EEG in awake moving animals was dominated by a theta rhythm of ∼ 8 Hz for both C57BL/6 (*n *= 6) and 129X1 (*n *= 4) mice (Fig.2A and C). Following administration of dexmedetomidine, the EEG from C57BL/6 animals showed a dominant delta oscillation (between ∼ 2 and 4 Hz) with a small shoulder at about 5 Hz. An averaged Fourier transform power spectrum is shown in Fig.2A for C57BL/6 mice that had lost their righting reflex. An averaged Morlet Wavelet transform is shown in Fig.2B after dexmedetomidine administration. At the moment of LORR, the delta peak increased in power and slightly reduced in frequency. For the 129X1 mice injected with the same dose of dexmedetomidine (400 μg/kg) during a comparable time period after injection, the EEG (Fig.2C and D) showed a marked peak at delta frequencies, and also a comparably strong band at theta frequencies (∼ 5–7 Hz). There was no LORR – the animals remained awake and continually adjusted their posture in the rotating cylinder.

### Controls for the 129X1 *Adra2a* gene

**Figure 3:**
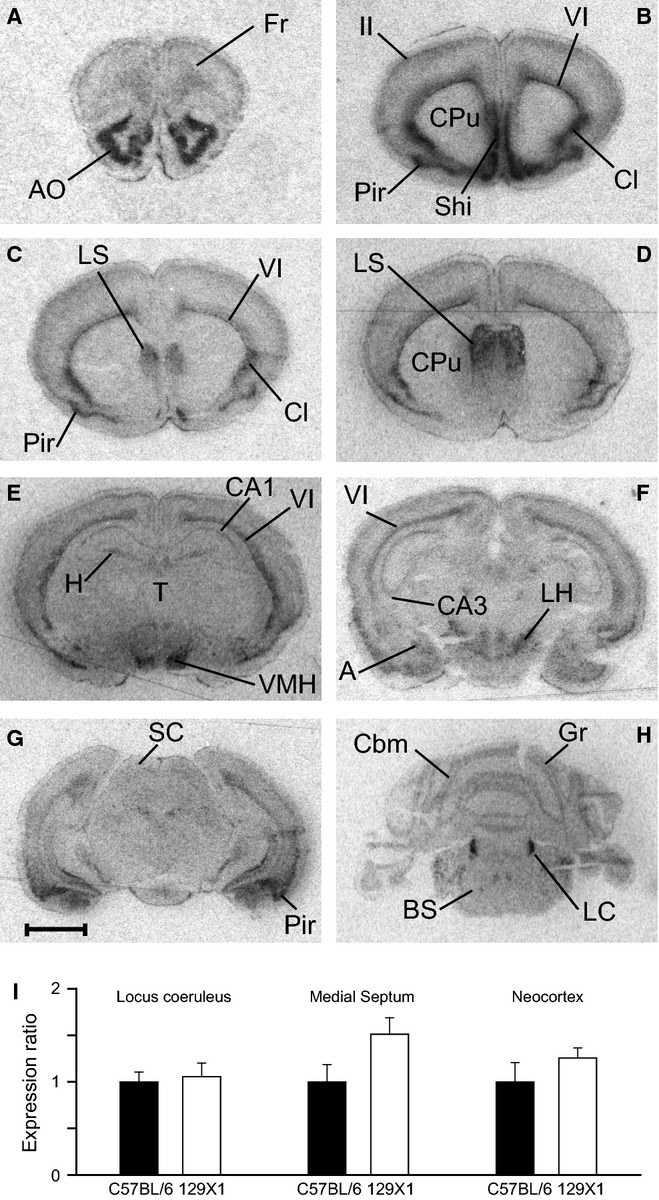
(A–H) *In situ* hybridization with a ^33^P-labelled oligonucleotide probe showing the distribution of *Adra2a* messenger RNA in coronal brain sections from 129X1 mice (*n *= 3). A, amygdala; AO, anterior olfactory nucleus; BS, brainstem; Cbm, cerebellum; Cl, claustrum; CPu, caudate-putamen; Fr, frontal cortex; Gr, granule cells; H, hippocampus; LC, locus coeruleus; LH, lateral hypothalamic area; LS, lateral septum; Pir, piriform cortex; SC, superior colliculi; Shi, septal hippocampal nucleus; T, thalamus; VMH, ventral medial hypothalamic area; ‘II’ and ‘VI’, layers of neocortex. Scale bar: 2 mm. (I) Expression levels of *Adra2a* mRNA assessed by real-time PCR do not differ (Student's *t*-test) between mouse strains (*n *= 6 for each strain) in the locus coeruleus (*P *= 0.77), the medial septum (*P *= 0.08) or the neocortex (*P *= 0.31).

We found that dexmedetomidine induces sedation and LORR in 129X1 mice if they are not stimulated in any way, suggesting that the α_2A_ receptor is functioning normally in this strain; nevertheless, given that the *Adra2a* gene is essential for dexmedetomidine-induced LORR (Lakhlani *et al*., 1997; Gilsbach *et al*., 2009), we undertook additional control experiments to make sure the *Adra2a* gene was unaltered in the particular 129X1 substrain of mice we were using. We first checked whether the gene sequence of the α_2A_ receptor differed between strains. Using PCR, we amplified the single *Adra2a* coding exon from 129X1 and C57BL/6 mice, and found their nucleotide sequences to be identical (to one another, and to the reference sequence in the ENSEMBL database; data not shown). We next analysed the expression of the *Adra2a* gene in 129X1 brains by *in situ* hybridization (Fig.3A–H). The *Adra2a* gene is expressed in restricted cell types throughout the neuroaxis – regions or nuclei with particularly strong expression including the anterior olfactory nucleus, layer VI of the neocortex, the claustrum, the lateral septum, the ventral medial hypothalamic area and the locus coeruleus. Our *in situ* hybridization data match those reported for *Adra2a* mRNA brain distribution for mice (Wang *et al*., 1996) and rats (Nicholas *et al*., 1993). Finally, using real-time PCR, we examined the levels of *Adra2a* gene expression in the locus coeruleus, medial septum and neocortex, and found no significant differences in expression levels in these regions between the two mouse strains (Fig.3I).

### The phenotype, ‘prevention of dexmedetomidine-induced LORR by a stimulus’, is a dominant-like trait

**Figure 4:**
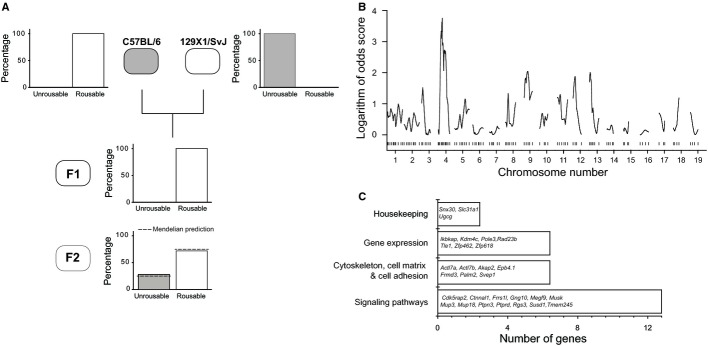
Genetic mapping shows that a region on chromosome 4 has a large influence on the ability of a stimulus to prevent dexmedetomidine-induced LORR in 129X1 mice. (A) Crossing C57BL/6 mice with 129X1 showed that the arousability from dexmedetomidine LORR behaves as a dominant-like trait (*n *= 18 mice from F_1_ generation). (B) Genome-wide linkage analysis indicates a region on chromosome 4 that has a major impact on the phenotype. (C) A summary of the 30 genes that might be responsible for the differences between the two mouse strains in the ability of a stimulus to reverse dexmedetomidine LORR.

We were interested in establishing the genetic reasons for the marked binary difference in dexmedetomidine-induced LORR between the inbred C57BL/6 and 129X1 mouse strains when the mice were receiving an arousal stimulus. We analysed adult progeny from F_1_ crosses of both male C57BL/6 with female 129X1, and female C57BL/6 with male 129X1 mice. All adult mice from both sets of crosses were insensitive to dexmedetomidine-induced LORR (2 mg/kg; *n *= 18; Fig.4A). This suggested that the ability of a stimulus to prevent dexmedetomidine-induced LORR was a dominant-like trait from the 129X1 mice and that the trait was not sex-linked. We then inter-crossed F_1_ progeny to generate the mapping F_2_ population, and screened all F_2_ mice (*n *= 55) for LORR at 400 μg/kg dexmedetomidine (i.p.), because this dose would guarantee LORR in C57BL/6 mice. From the F_2_ animals screened, 72% had no dexmedetomidine-induced LORR and 28% had a LORR, giving a ratio of 2.6 : 1, which was not significantly different (*P* > 0.99, Chi-squared test) from the 3 : 1 ratio predicted by a single locus model for a genetic determinant (Fig.4A). All the animals that were negative in this screen were screened for dexmedetomidine-induced LORR at 2 mg/kg and all were negative (data not shown). The F_2_ animals that did show LORR were backcrossed with C57BL/6 and the progeny screened for LORR at 400 μg/kg, all of which were positive. We then undertook a genetic mapping by genome-wide linkage analysis in the F_2_ progeny to determine whether a single major-effect locus allows stimuli to prevent dexmedetomidine-induced LORR, or if multiple loci combine to generate a threshold effect whereby only some loci are required to be co-inherited to cause the effect.

### Genetic mapping of the trait influencing the ability of a stimulus to prevent dexmedetomidine-induced LORR in 129X1 mice

Our permutation analysis with 10 000 permutations yielded a threshold LOD score value of 3.5 for a genome-wide significance of 5%. The genome-wide linkage analysis using the interval mapping method, assuming a binary trait on 55 F_2_ progeny, yielded a LOD score peak on mouse chromosome four flanked by the markers D4Mit108 and D4Mit139 with a LOD score (LOD = 3.8) exceeding the threshold level for significance at 95% confidence (Fig.4B). This is equivalent to a probability of 0.031 that the linkage being identified occurred due to chance. Linkage analysis also yielded two regions on chromosomes 9 and 13 with LOD scores ∼ 2 (Fig.4B). We then used the (parametric) MQM module, with markers D9Mit263 and D13Mit117 as co-factors. By using the co-factors as a way to control the genetic background and to absorb the genetic effects of their nearby QTLs, hence eliminating the phenotypic variation induced by unlinked QTLs, the LOD score peak (flanked by D4Mit108 and D4Mit139 markers) increased to 4.4 (not shown). Thus, our linkage analysis identified a major locus on chromosome 4 and two minor modifying loci on chromosomes 9 and 13 influencing the arousability from dexmedetomidine-induced LORR.

The confidence interval for the QTL region on mouse chromosome 4 (minus 1 LOD support interval) is located between 52 155 238 and 77 521 241 bp. This region contains 129 protein-coding genes, of which 26 have single nucleotide polymorphisms (SNPs) between the two strains (Fig.4C). In addition to SNPs, this region contains 67 copy number variations (CNVs; LINE, SINE, LTR and Simple Repeats) that are polymorphic between the two progenitor strains (http://variation.osu.edu/mouse_indel/index.html). Of the 26 SNP-bearing protein coding genes, seven genes, namely *Ikbkap* (inhibitor of kappa light polypeptide gene enhancer in B-cells), *Ugcg* (UDP-glucose ceramide glucosyltransferase), *Rgs3* (regulator of G-protein signalling 3), *Cdk5rap2* (CDK5 regulatory subunit-associated protein 2), *Megf9* (multiple EGF-like-domains 9), *Tle1* (transducin-like enhancer protein 1) and *Ptprd* (receptor-type tyrosine-protein phosphatase delta) also contain CNVs polymorphic between the two parental strains. In addition, four genes in the region contain only CNVs polymorphic between the two strains (Fig.4C). Finally, the minus 1 LOD support interval contains five non-coding RNA genes (*Smc20s*, *n-R5s185*, *Mir32*, *Mir3095*, *Mir455*), of which only *Smc20s* contains a SNP polymorphic between the two parental strains.

## Discussion

Inbred mouse strains, including of course the C57BL/6 and 129X1 strains used in this study, have many innate differences in behaviour and neurological profile, which by definition are determined genetically (Crawley *et al*., 1997; Logue *et al*., 1997). It is also established that the strains C57BL/6 and 129X1 have small differences in sensitivity to anaesthetics and hypnotics (Homanics *et al*., 1999). Surprisingly, as we report here, these two strains exhibit a binary difference in their ability to stay awake following administration of dexmedetomidine during an arousal-promoting activity (maintaining their posture in a rotating tube). The α_2_ adrenergic system, and in particular the agonist dexmedetomidine, provides an interesting angle to get at any specific mechanisms of how stimuli prevent sleep because α_2_ activation seems to induce a state resembling natural sleep (Mason *et al*., 2009). Patients and animals can be roused from a dexmedetomidine-induced state. Here we show that, on the converse side, this arousability has strong genetic determinants.

### Pharmacokinetic differences in response to dexmedetomidine are unlikely to explain the phenotype

In our study, the two mouse strains responded identically to low, sedative concentrations of dexmedetomidine; higher doses produced non-stimulus challenged LORR in both strains. This implies similar pharmacokinetics and similar adrenergic signalling pathways in both mouse strains. The cytochrome P450 superfamily of haemoproteins metabolize substances such as drugs, hence the type and amount of the P450 enzyme expressed determines the animal's metabolic response to the administered drug (Wrighton & Stevens, 1992). In rabbits and dogs, medetomidine (the racemic mixture of dexmedetomidine and its enantiomer levomedetomidine) is metabolized by the hepatic enzymes CYP2D, CYP2E and CYP3A (Avsaroglu *et al*., 2008; Duhamel *et al*., 2010). In mouse, the *Cyp2d* gene cluster that is homologous to rabbit *Cyp2d* is located on chromosome 15, whereas the *Cyp2e1* gene homologous to rabbit *Cyp2e* is located on chromosome 7 and the *Cyp3a* gene cluster homologous to the dog *Cyp3a* gene is located on chromosome 5. None of these three chromosomes in our mapping study yielded significant LOD scores, suggesting that the difference between the two strains is not likely due to differences in pharmacokinetics of dexmedetomidine.

### Strain differences in dexmedetomidine-induced hypnosis

If not drug kinetics, what could explain the ‘yes–no’ difference between the C57BL/6 and 129X1 mouse strains in the ability of a stimulus to maintain wakefulness in the presence of dexmedetomidine? Given that mice with deleted or global knocked down *Adra2a* gene have no dexmedetomidine-induced LORR (Lakhlani *et al*., 1997; Tan *et al*., 2002; Gilsbach *et al*., 2009), an obvious candidate would be changes in either sequence, expression level or expression pattern of the *Adra2a* gene itself (located on mouse chromosome 19); but none of these differed between the two strains, and chromosome 19 did not give a significant LOD score in the breeding experiments. Another possibility could be strain differences in the TASK1 (*kcnk3*) potassium channel gene, because we found that TASK1 knockout mice are less sensitive to low (30 μg/kg) sedative doses of dexmedetomidine (Linden *et al*., 2006); however, because the mouse *kcnk3* gene is on chromosome 5, again not a region that had a significant LOD score, we did not pursue this. Furthermore, as dexmedetomidine-induced sedation (as assessed by the Rotarod assay) and unstimulated LORR with dexmedetomidine are present in both the C57BL/6 and 129X1 mouse strains, this suggests that the α_2A_ adrenergic receptors and associated signalling are working similarly. Thus, we have to look beyond the *Adra2a* gene itself. One possibility would be a difference downstream of the α_2A_ adrenergic receptor that specifically affects LORR rather than sedation. Alternatively, it might be that the two strains differ in some arousal mechanism that may or may not involve the adrenergic system *per se*, but that is responsible for counteracting the inhibitory effects of dexmedetomidine. The phenotype could be explained, for example, by subtle differences in perception of stimuli. This could be caused by differences in expression of transcription factors during development, which affect the fine-scale patterning of circuitry. A clue might lie in the EEG. There is a clear difference in the EEG spectra between the two strains following a high dose (400 μg/kg) of dexmedetomidine – the C57BL/6 mice show a major peak at delta (2–4 Hz) frequencies with a shoulder at theta (4–7 Hz) frequencies (Fig.2A), whereas the 129X1 mice retain a markedly strong peak in the theta range (Fig.2C). The ability of the 129X1 mice to retain wakefulness, yet still exhibit a strong delta oscillation in the EEG, is not without precedent. For example, the sleep-promoting drug gaboxadol, a γ-aminobutyric acid (GABA)_A_ receptor agonist, causes increased delta oscillations in both the sleep and waking states (Winsky-Sommerer *et al*., 2007). The retained theta peak may well reflect the sensitivity of the 129X1 mice to a stimulus following dexmedetomidine administration. Agonists at α_2_ adrenergic receptors generate EEG oscillations with a component at theta frequencies that resemble the Type II ‘immobility’ theta emanating from the medial septum (Sainsbury & Partlo, 1991; Kitchigina *et al*., 2003; Wrzosek *et al*., 2009). Indeed, the α_2_ adrenergic receptor agonist clonidine directly injected into the medial septum generates a tuned theta oscillation in the EEG, possibly due to activation of α_2_ adrenergic receptors expressed on GABAergic neurons (Kitchigina *et al*., 2003). A hypothesis would be that dexmedetomidine causes both sedation (marked by delta oscillations) but also has an arousal component (as reflected by the theta oscillations), and it is this balance that gives dexmedetomidine its unique ability to provide arousable hypnosis. Interestingly, during non-REM sleep, a state that resembles dexmedetomidine-induced hypnosis, 129X1 mice have a significantly higher theta/delta ratio in the EEG spectrum than C57BL/6 mice (Huber *et al*., 2000), strengthening the view that dexmedetomidine-induced sleep and natural sleep share common neuronal pathways (Nelson *et al*., 2003).

### Mouse breeding and candidate genes

QTL analysis has been successfully used to identify the genes underlying variations in sleep architecture and EEG profiles in mice (Franken *et al*., 1998; Tafti *et al*., 2003; Maret *et al*., 2005; Andretic *et al*., 2008; Winrow *et al*., 2009), and we reasoned that a similar approach could be used here to investigate dexmedetomidine sensitivity. Data from LORR experiments indicated that the ability of a stimulus to prevent dexmedetomidine-induced LORR behaves as a dominant-like trait, and segregates to a region on chromosome 4 (LOD score = 4.4, MQM module). Precedents for dominant-like genetic loci determining complex brain behaviours in rodents are well established – the *tau* mutation in golden hamsters determines the period of circadian clock (Ralph & Menaker, 1988); a single dominant locus determines whether *Peromyscus* mice build an escape tunnel from their burrows (Weber *et al*., 2013); and after crossing C57Br/cdOrl and CBA/Orl mice, the duration of NREM and REM sleep episodes entirely resembled CBA mice (Valatx *et al*., 1972).

The region we identified on chromosome 4 that correlates with the mice being more resistant to dexmedetomidine-induced LORR contains 26 genes bearing SNPs and 11 genes with CNVs polymorphic between the two strains. These genes code for proteins involved in gene expression, signalling or the cytoskeleton (Fig.4C). Seven genes in this region contain both SNP and CNV polymorphisms between the progenitor strains. One of these genes, *Ikbkap*, encodes a transcription factor (Anderson *et al*., 2001), which when mutated produces familial dysautonomia disease, characterized by cell death in the autonomic nervous system (Anderson *et al*., 2001). SNPs polymorphic between the two parent strains are located in various regions of the *Ikbkap* gene; five polymorphic SNPs are located in the mRNA untranslated regions, 47 SNPs are within introns and five SNPs are in the coding regions. Of these five SNPs, three are non-synonymous, whereas the remaining two are predicted to cause amino acid changes. Another gene, located in the minus 1 LOD support, contains CNV polymorphisms between the two mouse strains. The gene, *Slc31a1* (solute carrier family 31, member 1) encodes a copper transporter (Kuo *et al*., 2001). Null mutations lead to defective embryonic growth and development, and reduced copper transport into the brain (Kuo *et al*., 2001). The CNV in the *Slc31a1* gene is a ‘long interspersed nuclear element’ (LINE), and is located in an intron. This LINE element is only present in the C57BL/6 strain.

In addition to the differences between protein-coding genes, differences in non-protein coding genes in the QTL region could also, in principle, produce the phenotype – a mutation in a microRNA gene, which in turn governs the expression levels of a protein-coding gene, or in some other non-coding genetic element, or in the extent of DNA methylation of the region could all contribute. The minus 1 LOD support interval that we determined in our study contains five non-coding RNAs of which only one, *Smc20*, contains a point mutation polymorphic between the two parental strains.

## Conclusion

As far as we know, there has been rather little work done on the genetic influences for how easily we fall asleep. In this study, as a surrogate for the induction of sleep, we used the adrenergic alpha2a receptor agonist dexmedetomidine, which has the intriguing property of allowing patients to be roused if given a sufficient stimulus such as gentle shaking. Clearly the state induced by dexmedetomidine, if not actually sleep, resembles nREM sleep in both the EEG profile induced and its sensitivity to external stimuli. In this paper, we showed that a strain of mice is resistant to the sleep-inducing properties of dexmedetomidine if there is sufficient sensory input and, surprisingly, this had a genetic basis. The definitive identification of the gene(s) involved will require fine mapping of the 25-Mbp region determined in our coarse mapping experiments, and additional specific genetic manipulation of several putative targets. This might ultimately shed light on some of the natural processes governing how various stimuli prevent the onset of sleep.

## Abbreviations

**Abbreviations**
CNV: copy number variation
EEG: electroencephalogram
GABA: γ-aminobutyric acid
LINE: long interspersed nuclear element
LOD: Logarithm of Odds
LORR: loss of righting reflex
MQM: Multiple-QTL-Mapping
NREM: non-rapid eye movement
PCR: polymerase chain reaction
QTL: Quantitative Trait Locus
REM: rapid eye movement
SNP: single nucleotide polymorphism

## Supporting Information

Data S1 Link to supplementary videos.
