# Supplementary material for: Staying awake – a genetic region that hinders α2 adrenergic receptor agonist-induced sleep
Source: Eur J Neurosci. 2014 Mar 27;40(1):2311–9. doi: 10.1111/ejn.12570 (PMC4215598; doi:10.1111/ejn.12570)
Supplement: Data S1 — Link to supplementary videos. [file ejn0040-2311-sd1.docx]

**Link to supplementary videos**

S1) a C57Bl/6 mouse immediately after the injection of 400 μg kg^-1^ dexmedetomidine

S2) 10 minutes later

S3) a 129X1 mouse immediately after the injection of 400 μg kg^-1^ dexmedetomidine

S4) 10 minutes later.

<https://www.dropbox.com/sh/jrh81s8nop4ajpy/k7ow8iyTA2>
